# Meta-Analysis: Identification of Low Birthweight by Other Anthropometric Measurements at Birth in Developing Countries

**DOI:** 10.2188/jea.JE20100182

**Published:** 2011-09-05

**Authors:** Eita Goto

**Affiliations:** Department of Medicine, Mizawa Hospital, Hamamatsu, Japan

**Keywords:** meta-analysis, low birth weight, anthropometry, sensitivity and specificity, newborn

## Abstract

**Background:**

Low birthweight should be identified early, even in developing countries where birthweight cannot be easily measured due to the absence of scales and trained staff. This meta-analysis evaluated and compared the use of other anthropometric measurements at birth to predict low birthweight.

**Methods:**

All studies of medium to high quality (Quality Assessment of Diagnostic Accuracy Studies score ≥8) published in English were included. Bivariate random-effects meta-analysis and hierarchical summary receiver operating characteristic curves were used.

**Results:**

A total of 69 studies evaluated foot length or the circumference of the chest, (mid-upper) arm, or thigh (*n* = 8, 25, 30, and 6, respectively). Chest circumference and arm circumference had areas under the curve >0.9 (0.95 for both), pooled positive likelihood ratios >5 (8.7 and 10.3, respectively), and negative likelihood ratios <0.2 (0.13 and 0.17, respectively); thigh circumference and foot length were less accurate. There was no substantial difference between chest and arm circumference with respect to pooled sensitivity (0.88 vs. 0.84, *P* = 0.505), specificity (0.90 vs. 0.92, *P* = 0.565), or diagnostic odds ratio (67 vs. 60, *P* = 0.552). However, as compared with arm circumference, chest circumference showed greater clustering of observations on the hierarchical summary receiver operating characteristic curve and narrower 95% confidence and prediction regions.

**Conclusions:**

Chest circumference and arm circumference have similarly high, although not confirmative, accuracy in predicting low birthweight; however, chest circumference appears to be more precise.

## INTRODUCTION

Low birthweight (<2500 g) is an important public health problem because it is associated with poorer outcomes than normal birthweight.^1^^,^^2^ Therefore, low birthweight should be detected early to allow newborns to receive appropriate care soon after delivery. However, in some developing countries where home delivery is fairly common despite the high prevalence of low birthweight, it may be difficult to measure birthweight because of inadequate equipment^3^^–^^13^ and a lack of trained health staff.^4^^,^^5^^,^^7^^,^^8^^,^^13^^,^^14^ In response to the demand for a rapid, simple, and reliable screening approach for low birthweight, other anthropometric measurements at birth have been studied as surrogates for birthweight, including chest circumference^6^^,^^8^^,^^10^^,^^11^^,^^14^^–^^16^ and (mid-upper) arm circumference.^3^^,^^4^^,^^7^^,^^9^^,^^10^^,^^12^^–^^19^ The number of participants in each of these studies, however, may have been too small to generalize the conclusions to the target populations.^7^^,^^16^ Furthermore, findings vary among studies, and controversy remains regarding the best surrogate measure. A previous meta-analysis found stronger correlations between birthweight and both chest and arm circumferences as compared with other newborn parameters, but did not evaluate the diagnostic performance of these measurements in predicting low birthweight.^20^ The present meta-analysis utilized pooled sensitivity, specificity, positive and negative likelihood ratios, and the diagnostic odds ratio, as well as hierarchical summary receiver operating characteristic curves, to compare the accuracy of other anthropometric measurements in identifying low birthweight.

## METHODS

### Primary outcomes

The primary outcomes were the sensitivity and specificity for predicting low birthweight by birth height; head, chest, (mid-upper) arm, abdominal, thigh, and calf circumferences; foot, sternal, sole, and crown-to-rump or crown-to-coccyx lengths; and subscapular and tricipital skinfold thicknesses.

### Selection criteria, search strategy, and data extraction

The selection criteria were: (1) studies published in English, (2) studies that investigated the diagnostic accuracy of other newborn anthropometric measurements at birth in predicting low birthweight, and (3) studies of high quality (ie, Quality Assessment of Diagnostic Accuracy Studies [QUADAS] score ≥8; see below). Using the Falck-Ytter filter,^21^ the PubMed database was searched to locate articles that displayed phrases for the abovementioned anthropometric outcomes in the title or abstract. Each time an article that included 1 or more studies satisfying the selection criteria was identified by scanning the title and abstract, other articles shown under “See all related articles (Related citations See all…)” on the right side of the web page were also scanned. Articles in the references of already collected articles were also evaluated. MEDLINE, EMBASE, CINAHL, PsychINFO, Wiley InterScience, ProQuest Medical Library, the entire Cochrane Library (eg, CENTRAL), and Google Scholar were also used repeatedly (June, 2010). Articles were not excluded due to date of publication. The true positive, false positive, false negative, and true negative values were extracted. When possible, any missing data were calculated by using other existing data, including number of participants, prevalence of low birthweight, and diagnostic indices.

### Quality assessment

Study quality was assessed using the QUADAS tool,^22^^,^^23^ which consists of 14 questions to assess the quality of studies investigating diagnostic performance. The total number of “yes” responses to 14 questions is referred to as the QUADAS score. The Standards for Reporting of Diagnostic Accuracy (STARD) checklist^24^ was also used to score studies in a similar manner. A study with a QUADAS score of 8 or higher was regarded as eligible, and studies with a QUADAS score greater than or equal to 10 were compared with those with a QUADAS score less than 10 in subgroup analysis. This was done because a QUADAS score of 8 or 10 is commonly regarded as high in meta-analyses published in the most prestigious and other journals,^25^^–^^28^ although neither the QUADAS score, which indicates high quality, nor the numerical methods used to generate scores is uniform.^25^^–^^28^

### Data analysis

A bivariate random-effects model was used to pool sensitivity, specificity, positive and negative likelihood ratios, and diagnostic odds ratio. Logit-transformed sensitivity and specificity (assumed to be normally distributed, correlated random effects) were integrated.^29^ The random-effects model allows for heterogeneity among studies. Summary sensitivity and specificity and the corresponding positive and negative likelihood ratios and diagnostic odds ratios were derived from the standard output of the bivariate model, ie, mean logit sensitivity and specificity with their standard errors and 95% confidence intervals (CIs) and the estimates of the between-study variability in logit sensitivity and specificity and the covariance between them. Hierarchical summary receiver operating characteristic curves were simultaneously constructed, and the areas under the curves were also calculated. The ideal cut-off points were derived from the Youden Index, defined as the point on the summary receiver operating characteristic curve that is the farthest from the straight line (representing “area under the curve = 0.500”) that passes through the origin with a 45° angle relative to the Y-axis.^30^ Heterogeneity was assessed using *I*^2^: a value of *I*^2^ > 50% was considered to indicate substantial heterogeneity. Sensitivity analysis was conducted to identify sources of heterogeneity in the process of selecting the studies depending on (a) “yes” only or (b) “yes” or “unclear” responses to each of the 14 items on the QUADAS. Publication bias was assessed using Deeks’ funnel plot asymmetry test.^31^ Subgroup analysis was conducted to assess whether pooled sensitivity or specificity significantly differed by certain study characteristics, namely, Asia vs. other regions, presence vs. absence of a 2 × 2 table, and a QUADAS score greater than or equal to 10 vs. a score less than 10. Stata/SE 11.1 (StataCorp) was used for all analyses.

## RESULTS

### Systematic review

**Figure 1.**
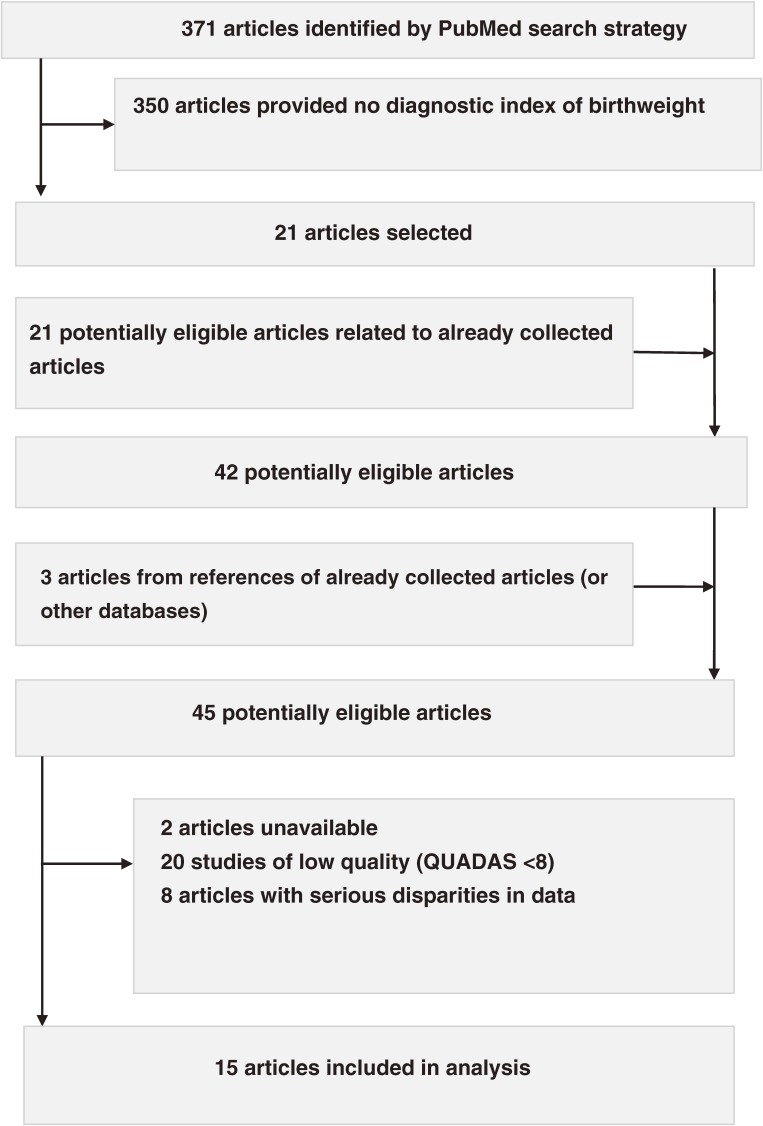
Flow diagram for selection of studies

**Figure 2.**
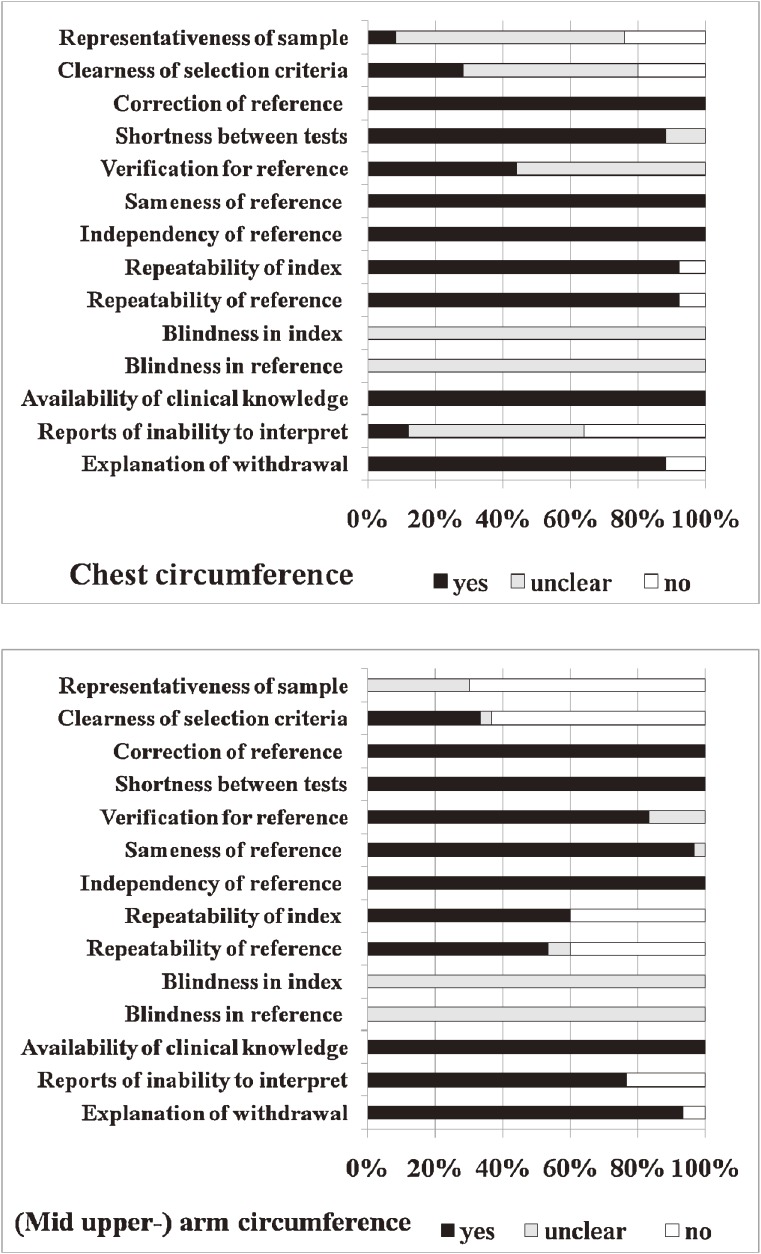
Summary of QUADAS quality assessment of included studies

The literature search and article references identified 45 articles that evaluated the use of other newborn anthropometric outcomes at birth to predict low birthweight (Figure 1). Among these, 2 articles were not available from libraries in Japan (the author’s home country), the British Library, or the US National Library of Medicine. Twenty studies were of low quality. Of the 15 articles (Table 1) that remained after excluding 8 studies with disparities in numerical values for diagnostic indices and/or 2 × 2 tables (Table 2), the author extracted 1 study evaluating birth height, 1 evaluating head circumference, 25 evaluating chest circumference, 30 evaluating arm circumference, 6 evaluating thigh circumference, 2 evaluating calf circumference, and 8 evaluating foot length.^3^^,^^4^^,^^6^^–^^19^ The study regions comprised Africa, Asia, Europe, and the Middle East, but not Latin or North America. In evaluating birth height; head, chest, arm, thigh, and calf circumferences; and foot length, the total number of participants was 703, 609, 37 293, 22 615, 1964, 624, and 13 120, respectively, while the total number of low-birthweight births was 121 (17%), 105 (17%), 7950 (21%), 3732 (17%), 476 (26%), 126 (20%), and 3752 (29%), respectively. The total number of positive results for the index test was 163 (23%), 219 (36%), 10 458 (28%), 5933 (24%), 453 (23%), 172 (28%), and 5472 (42%), respectively. No study was blinded to the index or reference test(s), but all studies chose the correct reference tests, measured the reference tests independent of the results of the index tests, and interpreted the tests by using the same clinical data as in practice (ie, interpretation of the tests was not affected by the clinical data) (Figure 2). Regardless of STARD score (range, 6/25 to 16/25), the included studies were limited to those with a QUADAS score of 8 or higher. Four studies evaluating chest circumference and 6 studies evaluating arm circumference were of very high quality (QUADAS ≥10) (Table 3) and were extracted from 4 articles (Table 1).

**Table 1.** Characteristics of studies that evaluated the performance of chest and arm circumferences as predictors of low birthweight

	Year	Region	Measurement	Cut-off point (cm)	Number of participants	Prevalence of LBW (%)	Prevalence of positive test result (%)	2 × 2 Table	QUADAS
Amhed et al	2000	Asia	MUAC	9	1676	27	12	No	8/14
Arisoy et al	1995	Europe	CHC	29.5 to 30.5	874	11	10 to 17	No	10/14
			MUAC	8.5 to 9.5	874	11	9 to 30	No	10/14
Das et al	2005	Asia	MUAC	9	456	34	35	Yes	10/14
Ezeaka et al	2003	Africa	MUAC	9.6^a^	701	18	26	No	9/14
Fok et al	2005	Asia	CHC	29.5, 29.9	5478, 4861	5, 6	13, 12	No	9/14
Hossain et al	1994	Middle East	MUAC	9 to 10	148	1	2 to 27	Yes	9/14
Huque et al	1991	Asia	CHC	30.14	217	41	37	Yes	11/14
			MUAC	8.9	217	41	33	Yes	11/14
Khanam et al	1990	Asia	MUAC	8.8	206	48	41	Yes	9/14
Kulkarni et al	1993	Asia	CHC	27.5, 28	312	20	18, 29	Yes	9/14
			MUAC	8.5, 9	312	20	27, 46	Yes	9/14
Mullany et al	2007	Asia	CHC	29.7 to 30.9	1640	29	25 to 48	No	8/14
Ngowi et al	1993	Africa	CHC	29.4	833	29	22	No	8/14
			MUAC	9.3	833	28	18	No	8/14
Ramji et al	1986	Asia	MUAC	8.4	216	36	30	Yes	10/14
Sachar et al	1994	Asia	MUAC	7, 8.5	281	14	1, 16	Yes	8/14
Singh et al	1988	Asia	CHC	29.5 to 30.5	446	40	38 to 51	No	8/14
			MUAC	8.5 to 9.5	446	40	29 to 67	No	8/14
Sood et al	2002	Asia	MUAC	8.3 to 9.2	1272	12	1 to 100	No	8/14

LBW, low birthweight; CHC, chest circumference; MUAC, mid-upper arm circumference; QUADAS, Quality Assessment of Diagnostic Accuracy Studies.

^a^Studies using cut-off points >9.6 cm and <9.6 cm were excluded because of the disparity between the value of 1 diagnostic index in the study vs. the value of this index calculated from the remaining diagnostic indices.

**Table 2.** Reasons for exclusion of studies

	Measurement	Reason for exclusion (disparity in the numerical values of the diagnostic indices or 2 × 2 tables)
Gozal et al (1991)	MUAC	The value of 1 diagnostic index in the study vs. the value of this index calculated from the other diagnostic indices.
Landicho et al (1985)	CHC, MUAC	The value of 1 diagnostic index in the study vs. the value of this index calculated from the other diagnostic indices.
Sharma et al (1986, 8, 9, 90)	CHC, MUAC	The 2 × 2 table calculated from the prevalence in LBW in the study vs. the 2 × 2 table calculated from the number of positive results for the index test in the study.
Sreeramareddy et al (2008)	CHC	The value of PPV or NPV in the study vs. the value of PPV or NPV from the 2 × 2 table calculated from the prevalence of LBW and sensitivity and specificity.
Virdi et al (2001)	CHC	The value of PPV or NPV in the study vs. the value of PPV or NPV from the 2 × 2 table calculated from the prevalence of LBW and sensitivity and specificity.

CHC, chest circumference; MUAC, mid-upper arm circumference; PPV, positive predictive value; NPV, negative predictive value; LBW, Low birthweight.

**Table 3.** Meta-analysis of the accuracy of chest and arm circumferences in diagnosing low birthweight, and subgroup analysis by study region, inclusion of 2 × 2 table, and study quality

Variable			Number of studies	AUC	Sensitivity		Specificity		PLR	NLR	DOR	
					Estimate (95% CI)	*P* value	Estimate (95% CI)	*P* value			Estimate (95% CI)	*P* value
CHC^a^	Total	—	25	0.95	0.88 (0.85–0.91)	0.505	0.90 (0.86–0.93)	0.565	8.7	0.13	67 (55–81)	0.552

MUAC^b^	Total	—	30	0.95	0.84 (0.69–0.93)	—	0.92 (0.83–0.96)	—	10.3	0.17	60 (44–82)	—

CHC	Asia	Yes	21	0.95	0.89 (0.86–0.92)	0.102	0.88 (0.84–0.91)	0.000	7.2	0.12	58 (50–69)	0.001
		No	4	0.97	0.83 (0.74–0.89)	—	0.97 (0.94–0.98)	—	25.2	0.18	141 (84–238)	—
	2 × 2 table	Yes	—		—	—	—	—	—	—	—	—
		No	22	0.95	0.89 (0.86–0.91)	—	0.89 (0.85–0.92)	—	8.3	0.13	66 (53–81)	—
	QUADAS ≧10	Yes	4	0.95	0.86 (0.80–0.90)	0.333	0.97 (0.94–0.98)	0.000	25.2	0.15	170 (100–289)	0.000
		No	21	0.95	0.89 (0.85–0.92)	—	0.88 (0.84–0.91)	—	7.4	0.13	58 (50–68)	—

MUAC	Asia	Yes	22	0.95	0.86 (0.64–0.95)	0.672	0.91 (0.76–0.97)	0.712	9.5	0.16	60 (41–88)	0.827
		No	8	0.95	0.82 (0.68–0.91)	—	0.93 (0.86–0.97)	—	12.2	0.19	64 (42–99)	—
	2 × 2 table	Yes	11	0.94	0.74 (0.46–0.91)	0.285	0.94 (0.86–0.97)	0.411	11.5	0.27	42 (17–103)	0.363
		No	19	0.95	0.88 (0.67–0.96)	—	0.90 (0.71–0.97)	—	8.7	0.13	65 (49–86)	—
	QUADAS ≧10	Yes	6	0.96	0.85 (0.72–0.92)	0.908	0.95 (0.90–0.98)	0.400	17.7	0.16	109 (47–250)	0.092
		No	24	0.94	0.84 (0.64–0.94)	—	0.91 (0.77–0.97)	—	8.9	0.17	51 (38–69)	—

AUC, area under the curve; CI, confidence interval; PLR, positive likelihood ratio; NLR, negative likelihood ratio; DOR, diagnostic odds ratio; CHC, chest circumference; MUAC, (mid-upper) arm circumference; QUADAS, Quality Assessment of Diagnostic Accuracy Studies.

^a^*I*^2^ (%) for sensitivity, specificity, and DOR are 95.0, 98.6, and 100, respectively. ^b^*I*^2^ (%) for sensitivity, specificity, and DOR are 97.9, 99.8, and 100, respectively.

### Meta-analysis

Both chest and arm circumferences had high sensitivity and specificity (Table 3) and satisfied the criteria for high diagnostic accuracy (ie, an area under the curve of 0.9 to 1.0)^32^ and strong diagnostic evidence (ie, a positive likelihood ratio >5 and a negative likelihood ratio <0.2).^32^ These estimates, however, did not demonstrate a confirmative level of accuracy (ie, a positive likelihood ratio >10 and a negative likelihood ratio <0.1).^32^ Thigh circumference and foot length did not satisfy the criteria for satisfactory diagnostic accuracy due to their lower positive likelihood ratios (18.9 and 3.4, respectively) and higher negative likelihood ratios (0.29 and 0.28, respectively). There were too few studies of good quality to evaluate other anthropometric measurements (ie, birth height and head and calf circumferences).

**Figure 3.**
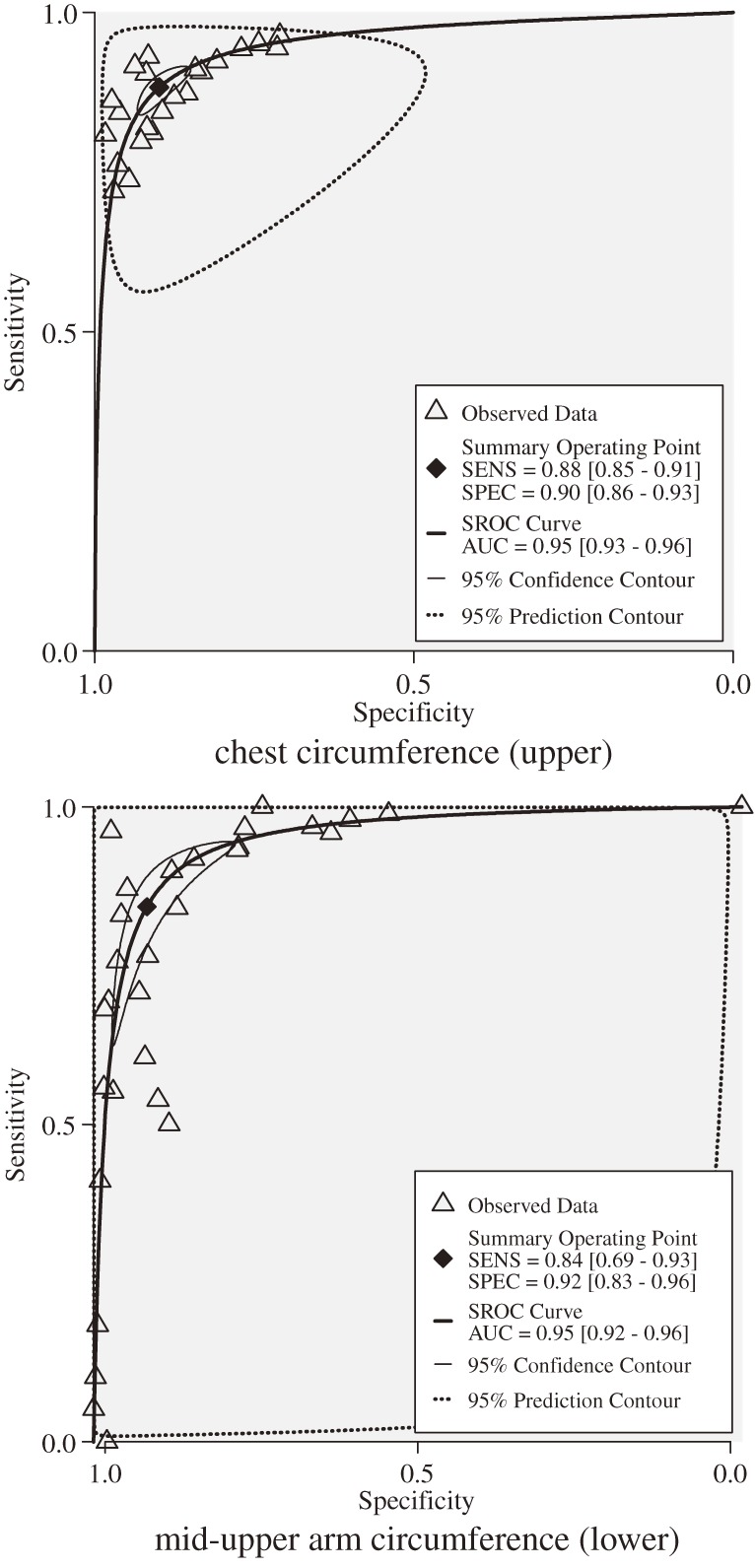
Hierarchical summary receiver operating characteristic curves and summary operating points of chest circumference (upper) and mid-upper arm circumference (lower). Abbreviations: SENS, sensitivity; SPEC, specificity; SROC, summary receiver operating characteristic; AUC, area under the curve

Neither sensitivity, specificity, nor diagnostic odds ratio statistically differed between chest and arm circumferences. There was marked heterogeneity (*I*^2^ ≥ 90%) for both chest and arm circumferences, thigh circumference (98%), and foot length (100%). The pooled estimates were not homogeneous in sensitivity analysis when only “yes” or “unclear”/“yes” responses were used for questions on the QUADAS or when potential outliers were omitted. On the hierarchical summary receiver operating characteristic curves, the observations for chest circumference were more clustered than those for arm circumference (Figure 3). The 95% confidence contour and prediction contour (ie, 95% of including the true sensitivity and specificity in a future study) for chest circumference were narrower than those for arm circumference. The most frequently used cut-off points for chest and arm circumferences were 30 cm and 9 cm, respectively, and these values were almost identical to the ideal cut-off points derived from the Youden Indices^30^ (Figure 3), as the pooled sensitivity and specificity when using chest circumference at cut-off points of 29.5 to 30.5 cm (sensitivity = 0.87 and specificity = 0.91) and using an arm circumference at cut-off points of 8.5 to 9.5 cm (sensitivity = 0.89 and specificity = 0.88) nearly corresponded to the sensitivity and specificity of the Youden Indices.

The Deeks’ funnel plot asymmetry test showed that the regression coefficients for assessing publication bias with regard to chest and arm circumferences were 16.4 (95% confidence interval [CI] −1.41, 34.3; *P* = 0.069) and −4.92 (−36.4, 26.2; *P* = 0.751), respectively. The criteria for both high accuracy and strong evidence of diagnostic performance were satisfied in almost all subgroups (Table 3). Additionally, within each subgroup, sensitivity, specificity, and diagnostic odds ratio rarely significantly differed between chest and arm circumferences: Asia (*P* = 0.647, 0.601, and 0.874, respectively) vs. other study regions (*P* = 0.884, 0.100, and 0.023, respectively) and a QUADAS greater than or equal to 10 (*P* = 0.861, 0.317, and 0.381, respectively) vs. less than 10 (*P* = 0.460, 0.601, and 0.463, respectively).

## DISCUSSION

To the author’s knowledge, this is the first meta-analysis of the predictive accuracy of other newborn anthropometric measurements at birth in diagnosing low birthweight. Because the results are highly dependent on the quality of the included studies, only studies of medium or high quality were included in the pooled diagnostic indices and the hierarchical summary receiver operating characteristic curves.

The number of articles increased to 45 after selecting 21 eligible articles via a PubMed search (Figure 1) because, even with the Falck-Ytter filter, which is a fairly dependable search strategy for PubMed,^21^ it was not possible to identify all potentially eligible studies. This indicated that when performing a diagnostic meta-analysis it is necessary (1) to investigate articles in the section headed “See all related articles (Related citations See all…)” on the right side of the PubMed web page when an eligible article was displayed on that web page, (2) to use search engines other than PubMed, and/or (3) to investigate citations in articles that have already been collected, whenever possible. Although blinding to the index or reference test(s), which is a likely cause of bias,^33^^–^^35^ was not done in any of the included studies, the use of the same reference test given for all participants regardless of the results of the index test (a more likely cause of bias than blinding^33^^,^^34^), the use of clinical populations rather than a diseased population plus a control group, and prospective data collection (the most likely cause of bias^33^^,^^34^) were confirmed in almost all the studies (98%, 100%, or 100% of studies, respectively).

Based on the present criteria, both chest and arm circumferences had high accuracy and strong evidence of diagnostic performance overall, although they may have lacked confirmative accuracy. Predictive accuracy appeared not to differ between these 2 measurements, as previously reported.^8^ Chest circumference, however, appears to be more precise and have less variability than arm circumference, as indicated by the narrower 95% confidence and prediction counters and the more densely gathered observations for chest circumference (Figure 3). The greater precision of chest circumference is a reason why its accuracy is more susceptible than that of arm circumference to confounding (eg, by study region and study quality; Table 3). This notable difference in precision^16^ is due at least in part to the larger measurement values^8^^,^^14^ and, possibly, lower elasticity of chest circumference. The interval in cut-off points among studies was identical, ie, 3 cm, between these 2 measurements (Table 1), and there was little difference in study quality between them (Figure 2). Average sample size in studies of chest circumference (*n* = 25) was smaller than in studies of arm circumference (*n* = 30). The variations in accuracy in different study groups suggest that extrapolation of overall pooled estimates to individual regions may not always be possible.^16^ The Deeks’ funnel plot asymmetry test showed absence of publication bias with regard to both chest and arm circumferences, while the inclusion of a sufficient numbers of studies allowed for the statistically significant standard of a *P* value of less than 0.05, as formally determined (*n* > 20).^36^ The test results must be interpreted with caution, however, particularly because the diagnostic odds ratios were very heterogeneous.^31^^,^^37^

The frequently used cut-off points for chest and arm circumferences are in accordance with the Youden indices^30^ (Figure 3). In this meta-analysis, however, the cut-off points varied considerably among studies, which could decrease predictive performance if cut-off points outside the appropriate ranges are used. The quality of diagnostic evidence when using chest circumference was strong, whether the cut-off points were within the range of 29.5 to 30.5 cm (positive and negative likelihood ratio = 9.9 and 0.14, respectively) or outside it (positive and negative likelihood ratio = 6.3 and 0.10, respectively), based on criteria for strong diagnostic evidence.^32^ However, diagnostic evidence when using arm circumference was not strong with cut-off points outside the range of 8.5 to 9.5 cm (positive and negative likelihood ratio = 26.3 and 0.51, respectively), although it was strong with cut-off points within that range (positive and negative likelihood ratio = 7.5 and 0.13, respectively). These findings are additional evidence of the superiority of chest circumference over arm circumference.

Identifying differences in diagnostic performance between studies that did and did not evaluate premature babies is an important goal. This was not possible in the present meta-analysis, however, because of the lack of eligible studies of chest circumference that explicitly excluded premature babies (*n* = 1) and the insufficient number of studies of arm circumference that explicitly enrolled premature babies (*n* = 3). Four or more such studies were needed for bivariate diagnostic meta-analyses.

### Strengths and weaknesses

This meta-analysis has the following strengths. First, the findings are likely to be generalizable due to the large number of included studies (*n* = 25 or 30). An extensive literature search was performed by investigating links to related articles on PubMed pages and by using multiple search engines. In addition, true positive, false positive, false negative, and true negative values were extracted whenever possible, even when data were not complete. In addition, large-scale studies were analyzed, including 1 study with as many as 5478 participants.^6^ The studies encompassed populations in Africa, Asia, Europe, and the Middle East, and the population was thus likely to be racially mixed. Second, bivariate random-effects meta-analysis was used to generate informative estimates. This bivariate model incorporates the correlation between sensitivity and specificity (more accurately, the correlation between logit-transformed sensitivity and specificity), which is not usually investigated.^29^ Additionally, 2-dimensional 95% prediction contours, which are also not utilized in conventional analyses, were used in addition to summary operating points (Figure 3). Third, the large number of included studies enabled subgroup analysis of study region and study quality as confounders. Fourth, the Deeks’ funnel plot asymmetry test was used to assess publication bias. This test yielded clearer results than the Begg, Egger, or Macaskill tests in meta-analyses of diagnostic accuracy^31^ because the diagnostic odds ratios were fairly high, there were thresholds representing the trade-off between sensitivity and specificity, fewer low birthweight infants were born than infants of normal birthweight, and substantial heterogeneity was observed. Finally, the estimates in this meta-analysis were backed by strong pooled correlations of birthweight with chest and arm circumferences (*r* = 0.84 and 0.81, respectively), which were calculated from a large number of studies (*n* = 71 and 76, respectively).^20^

There were also some weaknesses in this meta-analysis. First, the STARD scores of the included studies were generally not high. However, the sources of bias that would likely have the greatest impact on the results (ie, use of a clinical population rather than a diseased population plus control group, prospective data collection, and use of the same reference test for participants regardless of the results of the index test) were almost always controlled for, although a less important cause of bias (ie, blinding to the index or reference test) was present. Furthermore, meta-regression suggested that studies of higher quality (ie, QUADAS ≥10) substantially improved the sensitivity and diagnostic odds ratio of chest circumference (*P* < 0.001; Table 3). Therefore, the inclusion of more studies of high quality would be more supportive of the diagnostic accuracy of chest circumference. Second, there remains the possibility that relevant studies were not identified despite the use of varied search strategies. In addition, the authors of the identified studies were not contacted to obtain raw data in cases of missing or apparently erroneous data. The Deeks’ funnel plot asymmetry test, however, showed no evidence of publication bias. Additionally, studies were included even if they had slight data disparities, although the border between permissible and impermissible disparities was unclear. Third, sensitivity analysis did not eliminate most of the marked heterogeneity. Subgroup analysis, together with meta-regression, identified confounders as potential sources of heterogeneity, but heterogeneity was not sufficiently reduced after controlling for these confounders. On the other hand, this meta-analysis evaluated the performance of screening tests in primary care settings; therefore, the characteristics of the population must have varied (ie, must have been heterogeneous) in contrast to a diseased (ie, more homogeneous) population undergoing diagnostic tests in secondary or tertiary care settings. Fourth, conclusions drawn largely from hospitals or research centers were probably overestimated in cases of home deliveries for which lay people had to make the anthropometric measurements.^11^ Finally, the results might not be applicable to subgroup analyses of male vs. female, preterm vs. full term, singleton vs. nonsingleton, or appropriate-for-gestational-age vs. small-for-gestational-age infants.

### Conclusion

This meta-analysis used data from studies of medium to high quality to evaluate the identification of low birthweight by other anthropometric measurements. It was possible to pool the diagnostic indices for chest, arm, and thigh circumferences, and foot length; however, good-quality studies of other measurements were lacking. In sum, both chest and arm circumferences appear to have high accuracy and strong evidence of diagnostic performance in identifying low birthweight, and there was no substantial difference in accuracy between these two measurements. In contrast, thigh circumference and foot length were less accurate. According to hierarchical summary receiver operating characteristic curves, chest circumference was more precise than arm circumference; therefore, health workers and policy makers may favor this measure over arm circumference.
